# Labour market status and mortality risk: The Finnmark cohort study 1987–2017

**DOI:** 10.1177/14034948231174668

**Published:** 2023-05-19

**Authors:** Monika Dybdahl Jakobsen, Tonje Braaten

**Affiliations:** 1 Centre for Care Research North, UiT The Arctic University of Norway, Tromsø, Norway; 2 Department of Community Medicine, UiT The Arctic University of Norway, Tromsø, Norway

**Keywords:** Mortality, labour market status, health equity, Norway

## Abstract

**Aims:**

The aim of this study was to investigate the age-varying mortality risk associated with different labour market status categories.

**Methods:**

Data from a population-based survey carried out among adults aged 30–62 years in Finnmark in 1987/1988 were linked to the Norwegian Cause of Death Registry to identify all deaths occurring by December 2017. We used flexible parametric survival models to examine the age-varying associations between different labour market status categories (no paid work/homemaker, part-time work, full-time work, unemployment benefits, sick leave/rehabilitation allowance, and disability pension) and mortality.

**Results:**

Men with part-time work, unemployment benefits, sick leave/rehabilitation allowance, or disability pension had an increased mortality risk compared with men with full-time work; however, these findings were restricted to ages below 60–70 years, varying with labour market status category. For women, excess mortality was linked to disability pension in the younger age groups; in older age groups it was linked to the labour market status category no paid work/homemaker. Non-employment was associated with low education level compared with full-time employment.

**Conclusions:**

**The study showed increased mortality risk for some non-employment categories, with decreasing relative risk with age. Our findings suggest that the increased mortality risk is partly explained by health, pre-existing illnesses, and health-related behaviour and partly by other factors, such as social network and economic factors.**

## Introduction

The labour market is increasingly fragmented, and people have varying degrees of attachment to the labour market [1, 2]. Those who are in the labour force occupy different positions on a core-periphery axis, on which full-time employment with a permanent work contract and long-term unemployment represent the end points [2]. Those who are outside the labour force are also called the ‘inactive population’, and include labour market status (LMS) categories such as student, homemaker, and disability pensioner [3].

Studies suggest an association between low socioeconomic status and non-employment [4–6], and studies also show that individuals who belong to certain non-employment LMS categories, such as unemployed persons, persons with long-term sickness absence, and persons on disability pension, have an increased mortality risk compared with full-time employees [7–13]. Different, not mutually exclusive, explanations for the increased mortality risk have been suggested: One possible explanation is that poor health is incompatible with the demands of a job, and that people with poor health and pre-existing illnesses are more likely to become and remain non-employed [7]. Another explanation is that individuals cope with non-employment by changing their behaviour in unhealthy ways [7]. In addition, it has been theorised that non-employment leads to stress and social marginalisation, and has economic and social consequences that contribute to increased mortality [14, 15].

Studies suggest that LMS category at young age is associated with mortality risk in older age and that the increased mortality risk in certain non-employment groups is moderated by age. A systematic review showed that unemployment was associated with 73% increased mortality risk for people under the age of 40 years and 77% increased mortality risk for those who were in the age group 40–50 years [7]. However, this association was reduced after the age of 50 years [7]. Studies also suggest that persons who are granted disability pension in young age have higher mortality risk than persons who are granted disability pension in older ages [10, 16].

A considerable amount of research has investigated the association between LMS categories and mortality risk. However, few studies have investigated the age-varying associations between LMS categories and mortality. In the present study, we focus on multiple LMS categories, with the aim of investigating the age-varying mortality risk associated with different LMS categories.

## Materials and methods

### Data sources

The present analysis uses data from the third Finnmark County Health Survey, which was conducted by the National Health Screening Programme from March 1987 to June 1988 [17, 18]. Data from this health survey were linked to the Norwegian Cause of Death Registry to identify all deaths that occurred as of 1 December 2017. All residents of Finnmark County aged 40–62 years, as well as a 20% representative sample of residents aged 30–39 years, were invited to participate. Participants attended a physical examination (cholesterol level, blood pressure, serum lipids, body mass index) and responded to three self-administered questionnaires [17, 19]. The first questionnaire was sent along with the invitation letter, the second was given to participants at the time of the physical examination to be returned by surface mail, and the third was sent to all invitees 3 weeks after the examination. The questionnaires included questions on sociodemographic factors, employment, health conditions, physical activity, and diet, and were available in the Norwegian and Sami languages [17, 19]. Eighty-one per cent of invited men and 88% of invited women attended the physical examination [17]. All those who attended the physical examination also responded to questionnaire 1; 73% and 79% of them also responded to questionnaires 2 and 3, respectively [17]. Four hundred and forty-eight participants did not attend the physical examination but responded to one or more questionnaires. The study sample included participants who had attended the physical examination and/or had responded to one or more questionnaires, 17,554 participants, 8928 men and 8603 women.

All participants gave informed consent. The study was approved by the Regional Committee for Medical and Health Research Ethics (REC) North (2018/722) and the Norwegian Centre for Research Data was notified of the study (794054).

### Statistical analyses

The LMS category of each participant was determined based on the following questions: ‘Have you, in the last year, had: full-time work, part-time work, no paid work?’ (questionnaire 3), ‘Is homemaking your main job?’, ‘Do you have a full or partial disability pension?’, ‘Are you currently on sick leave or rehabilitation allowance?’, and ‘Have you, in the last 12 months, received unemployment benefits?’ (questionnaire 1). The replies to these questions were used to create the following LMS categories: (1) no paid work/homemaker; (2) part-time work; (3) full-time work; (4) unemployment benefits; (5) sick leave/rehabilitation allowance; and (6) disability pension. Among the participants, 22.9% had ticked off for more than one LMS category, and most of these (22.5%) were possible combinations such as part-time work and partly disability pension. In addition, 0.4% had ticked off for both full-time work and disability pension, which could be a result of a transition from full-time work to disability pension during the last year before the baseline. When more than one LMS category was applicable, the participant was placed in the category with the highest number on the list above – for example, those who ticked off for full-time work and on sick leave were classified as on sick-leave. We chose to prioritise disability pension over unemployment benefits and sick leave/rehabilitation allowance, because individuals most commonly transition from unemployment benefits or sick leave/rehabilitation allowance to disability pension [20].

We imputed a median baseline date of 1 November 1987 for the 448 participants who completed one or more questionnaires but did not attend the physical examination. Potential interactions between LMS category and marital status, LMS category and education level, and marital status and education level, were tested by including product terms.

Included covariates were education level, marital status (married, unmarried), self-rated health (excellent, good (ref.), fair, poor), smoking (never (ref.), former<median, former⩾median, current<median, current⩾median), and alcohol consumption. Education level was based on years of completed schooling, and classified differently in the younger and older age groups due to considerable differences in educational opportunities (<50 years of age: 0–8 (low), 9–12 (medium), ⩾13 (high); ⩾50 years of age: 0–7 (low), 8–9 (medium), ⩾10 (high)), in line with a classification used by Fylkesnes et al. [13]. We also conducted separate analyses to determine whether other classifications of education level influenced the regression coefficients. Median smoking was calculated based on a question concerning the number of cigarettes smoked per day. Alcohol consumption was based on the question ‘In the last year, how often have you drunk the equivalent of at least 5 half-bottles of beer, one bottle of wine, or ¼ bottle of liquor?’ Possible answers were ‘never’ (ref.), ‘a few times’, ‘1–3 times a month’, ‘1–2 times a week,’ and ‘3 or more times a week’. The covariates body mass index, physical activity, and place of residence were associated with a less than 10% change in the regression coefficients and were not included in the analyses.

Data were analysed using Stata version 16. We described participant characteristics by LMS category and distribution of LMS categories by education level and sex. We computed the overall crude mortality rate, as well as the crude and age-adjusted mortality rates by LMS category, using participants’ baseline age adjusted to the European standard population (2013).

Preliminary analyses with Cox proportional hazards regression models were carried out to assess the proportional hazards assumption and which variables to include in the flexible parametric survival (FPS) model. The proportional hazards assumption was assessed graphically, tested with Schoenfeld residuals and tested by adding a term for interaction between time and LMS category to the model. All these assessments indicated that the assumption was violated for LMS category, which suggested that the associations between LMS categories and mortality vary with age.

The graphical assessment and Schoenfeld residuals test also indicated that the assumption was violated for self-rated health in women. A model including a term for interaction between time and self-rated health was tested, and the test was significant for women (*P* = 0.0056), while it was borderline significant for men (*P* = 0.0516). A comparison of models (Akaike’s and Bayesian information criterion), indicated that stratification by self-rated health gave a better model fit than inclusion of self-rated health as a time-dependent variable.

After these initial analyses, we used FPS models to estimate hazard ratios [21]. We used attained age as the timescale because the present study is an observational study with age as a strong determinant of mortality risk [22, 23]. Separate analyses were carried out for men and women, because we could not rule out sex differences in recruitment to certain LMS categories, such as no paid work/homemaker and part-time work. Only participants with complete data (i.e. replies to all questions on LMS category and considered covariates) were included in the FPS model.

**Figure 1.**
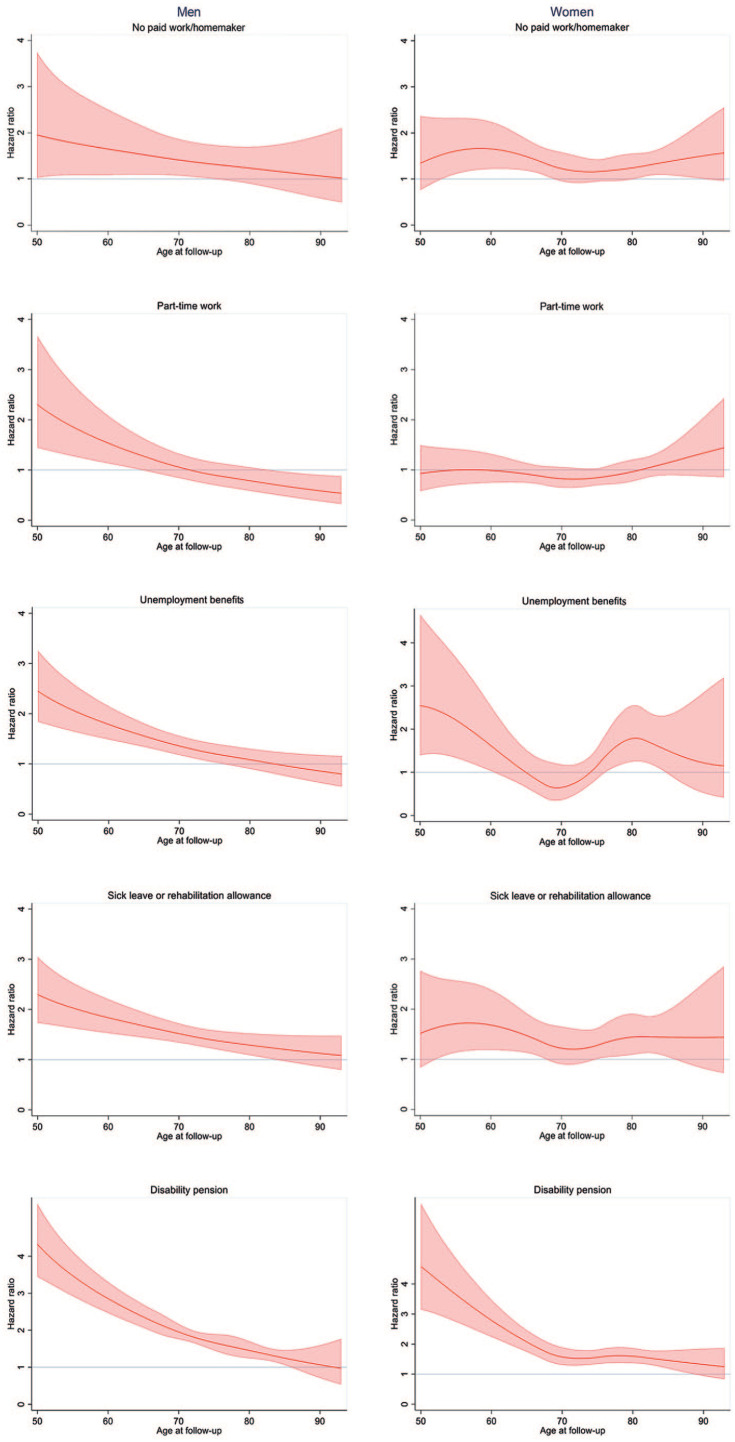
Mortality hazard ratios by labour market status category expressed as age-varying hazard ratios and 95% confidence intervals (reference: full-time work).

**Figure 2.**
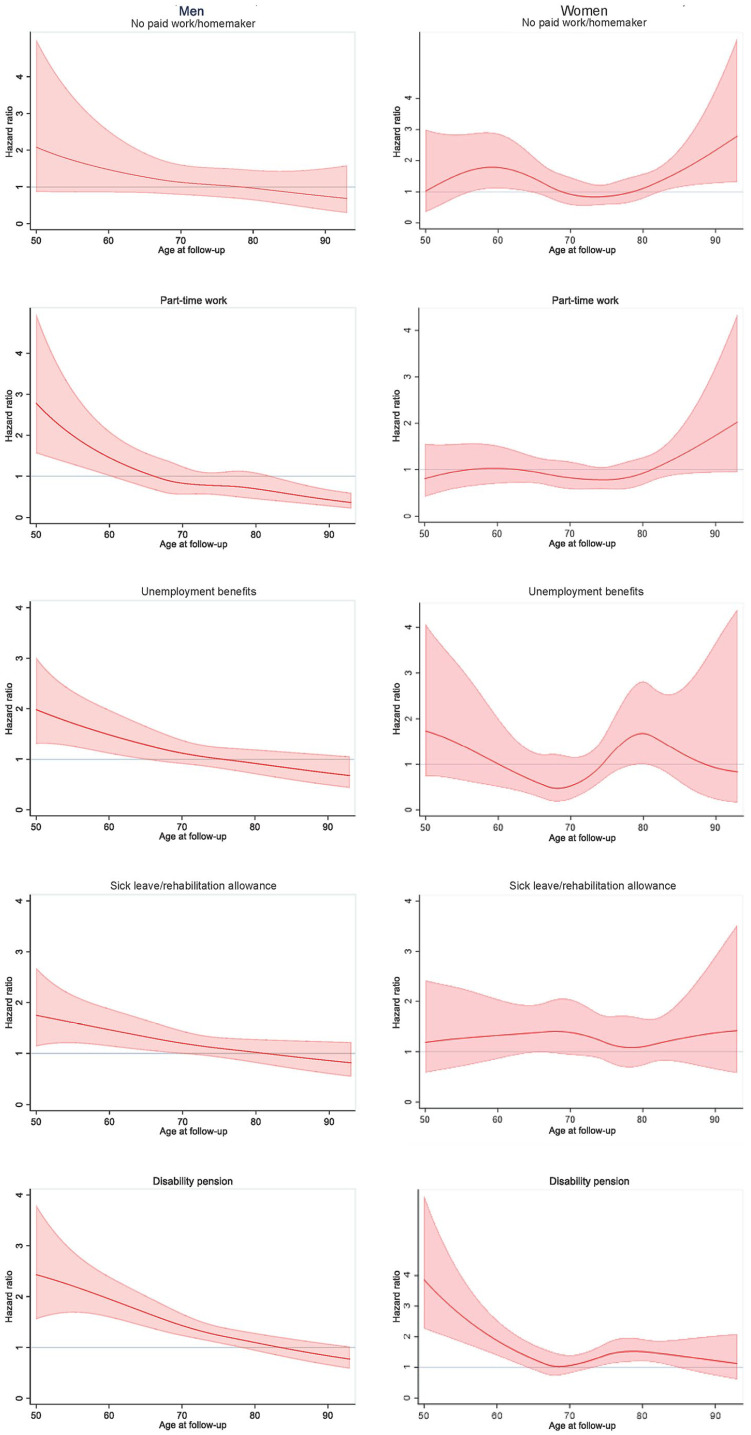
Mortality hazard ratios by labour market status category expressed as age-varying hazard ratios and 95% confidence intervals, controlled for education level, marital status, self-rated health, smoking and alcohol consumption (reference: full-time work).

The age-varying mortality risk associated with different LMS categories was modelled using the stpm2 command in Stata [24]. Based on Akaike’s and Bayesian information criterion, three degrees of freedom was chosen for the age-varying effect for both men and women, while four and two degrees of freedom were chosen for women and men, respectively, for the cumulative baseline hazard. After running stpm2, we created a temporary time variable and saved the corresponding predicted hazard ratios for each LMS category, and these variables were used to create line plots [24]. While all analyses included the whole sample (aged 30–62 years), the line plots did not include persons aged 49 years or younger, because few deaths were registered before the age of 50 years. If persons aged 49 years and younger had been included, this would have resulted in low statistical power and wide confidence intervals (CIs), which again would have led to a wide range on the y-scales, making the plots less visible. We presented mortality by LMS category expressed as age-varying hazard ratios, both without (Figure 1) and with the relevant covariates included in the model (Figure 2).

Finally, we conducted two sensitivity analyses. One was a comparative analysis, in which we compared the characteristics of participants with complete data for the variables included in the FPS model to those with missing data on these variables. Moreover, to investigate the possibility of distortion of associations due missing data, we compared the estimated mortality hazard of participants with complete data for the variables included in the FPS model with those with data on sex, age, marital status, LMS category, and smoking.

## Results

The mean age of participants was 48.0 years, and the distribution of participants by LMS category and mean age of each LMS category is presented in Table I. Among participants with full-time work, 23% had low education level, while this was the case for 68% of those with a disability pension, 46% of those with no paid work/homemakers, 46% of those with unemployment benefits, and 42% of those on sick leave/rehabilitation allowance. Accordingly, 41% of those will full-time work had high education, compared with 7% of those with disability pension, 12% of those with unemployment benefits, 14% of those with no paid work/homemakers, and 17% of those on sick leave/rehabilitation allowance (Table II).

**Table I.** Distribution of participants and mean age by labour market status category at baseline 1987/1988.

	*N*	%	Mean age
No paid work/homemaker^a^	1081	6.2	50.1
Part-time work	1730	9.9	48.0
Full-time work	7425	42.3	46.6
Unemployment benefits	848	4.8	47.3
Sick leave/rehabilitation allowance	1222	7.0	49.2
Disability pension	2871	16.3	54.8
Did not answer employment-related questions	2377	13.5	46.6

a 946 (87.5%) of these reported being a homemaker.

**Table II.** Labour market status category by education level, sex, and marital status at baseline 1987/1988.

	No paid work/homemaker *N* (%)	Part-time work *N* (%)	Full-time work *N* (%)	Unemployment benefits *N* (%)	Sick leave/ rehabilitation allowance *N* (%)	Disability pension *N* (%)
Education level^a^						
Low	312 (45.7%)	381 (29.8%)	1268 (23.2%)	258 (45.8%)	339 (41.5%)	1262 (67.7%)
Medium	274 (40.1%)	566 (44.3%)	1973 (36.0%)	237 (42.0%)	341 (41.7%)	475 (25.5%)
High	97 (14.2%)	330 (25.9%)	2233 (40.8%)	69 (12.2%)	137 (16.8%)	127 (6.8%)
Sex						
Men	105 (9.7%)	193 (11.2%)	4867 (65.6%)	508 (60.1%)	598 (49.1%)	1187 (41.5%)
Women	975 (90.3%)	1537 (88.8%)	2552 (34.4%)	337 (39.9%)	620 (50.9%)	1677 (58.5%)
Marital status						
Married	841 (77.9%)	1417 (82.0%)	5579 (75.2%)	521 (61.5%)	845 (69.2%)	1785 (62.2**%**)
Unmarried	239 (22.1%)	312 (18.0%)	1842 (24.8%)	326 (38.5%)	376 (30.8%)	1086 (37.8%)

a <50 years: 0–8 (low), 9–12 (medium), ⩾13 (high); ⩾50 years: 0–7 (low), 8–9 (medium), ⩾10 (high)),

The mean observation time was 25.8 years, and a total of 443,086 person-years were observed. Of the 17,554 participants in the study sample, 6864 died between 1987 and 2017 (39% of the entire sample, 45% of men, and 33% of women). The crude all-cause mortality rate was 15.5 per 1000 person-years, 18.4 for men and 12.3 for women. The age-adjusted mortality rate for men with full-time work was 18.3 (Table III), while the age-adjusted mortality rates for other LMS categories varied from 20.8 (part-time work) to 28.3 (disability pension). The age-adjusted mortality rate for women with full-time work was 12.5, while the age-adjusted rates for other LMS categories varied from 12.3 (part-time work) to 19.3 (disability pension).

**Table III.** Crude and age-adjusted mortality rates by labour market status category.

Men	Crude, %	Age-adjusted rate, %
No paid work/homemaker	57.1	21.2
Part-time work	44.6	20.8
Full-time work	36.8	18.3
Unemployment benefits	49.0	22.9
Sick leave/rehabilitation allowance	55.0	23.0
Disability pension	78.5	28.3
Women		
No paid work/homemaker	38.8	15.5
Part-time work	24.5	12.3
Full-time work	22.1	12.5
Unemployment benefits	28.0	15.0
Sick leave/rehabilitation allowance	33.5	15.7
Disability pension	58.3	19.3

The line plots showed that the estimated mortality risk was higher for part-time work, sick leave/rehabilitation allowance, unemployment benefits, and disability pension when compared with men with full-time work (Figure 1). However, these findings were restricted to age groups below 60–70 years, varying with LMS category. The mortality hazard ratios were lower but remained significant for sick leave/rehabilitation allowance, unemployment benefits and disability pension after inclusion of the covariates (Figure 2). Hazard ratios for men aged 50 years were 2.79 (95% CI 1.57–4.95) for part-time work, 2.43 (95% CI 1.56–3.80) for disability pension, 1.98 (95% CI 1.31–3.01) for unemployment benefits, and 1.75 (95% CI 1.14–2.68) for sick leave/rehabilitation allowance. The estimated hazard ratio decreased with age for men without full-time work, when compared with those with full-time work. There were few men with the LMS category no paid work/homemaker (Table II), and there were no statistically significant findings for men within this category compared with the reference group.

For women in the youngest age group, the estimated mortality risk was considerably higher for those with disability pension than for those with full-time work (Figure 1). The mortality hazard ratios were lower but remained significant after the inclusion of covariates (Figure 2). The hazard ratio for women aged 50 years with disability pension was 3.86 (95% CI 2.26–6.61). The hazard ratio was considerably lower for women aged 64 years with disability pension (1.38, 95% CI 1.02–1.87), and at age 65 years, there was no significant, increased mortality risk in women with disability pension compared with those with full-time work. The hazard ratios for women with no paid work/homemaker were above 1 in the oldest age groups, compared with full-time employees (e.g. hazard ratio 1.65, 95% CI 1.15–2.38 for women aged 85 years). Few women had the LMS category unemployment benefits (Table II), and there were no statistically significant findings for this group.

Comparative analyses showed that those with complete data for the FPS model differed from those without complete data in a number of respects. Among others, fewer women than men, fewer in the oldest and youngest age groups, and fewer participants from certain municipalities that responded had complete data. Our investigation of potential distortion of mortality risk estimates rendered only small differences.

## Discussion

The aim of the study was to investigate the age-varying mortality risk by LMS category, and we observed clear age and sex differences in mortality risk. Excess mortality in men was substantial for most LMS categories when compared with full-time work, but this excess was restricted to the youngest age groups. For women, however, substantial excess mortality was linked to disability pension in the younger age groups, and for older age groups it was linked to the LMS category no paid work/homemaker. Non-employment was associated with low education level compared with full-time employment.

The excess mortality in certain non-employment groups may have different explanations. In the present study, we adjusted for confounders such as self-rated health, education level, smoking, and alcohol consumption, which resulted in lower hazard ratios. This indicated that health and pre-existing illnesses, education level, and/or health-related behaviour partly explain the increased mortality rate. However, the hazard ratios for certain LMS categories remained significant after the inclusion of covariates, which suggest that other factors also may influence mortality. This is in line with Wallman et al. [16], who observed an increased mortality rate, particularly among young individuals with disability pension, which was not explained by underlying diseases, education level, or health-related behaviour. Wallman et al. [16] concluded that other factors, such as social network, economic factors, and the disability pension per se, may have detrimental effects on health. This assertion is supported by a systematic meta-review which concluded that employment can be beneficial to employees’ mental health, in particular if there are favourable workplace conditions [25]. However, to be able to draw conclusions about to what degree different explanation mechanisms have contributed to increased mortality risk, a different study design, with information on participants’ LMS categories and health over time, would be necessary.

The age difference in excess mortality that we observed is in line with findings from other studies [7, 14, 16], and may have different and complex explanations. Having a health condition at a young age may increase the risk of both being out of work and dying at a young age. Furthermore, previous studies have indicated that younger persons who experience unemployment can be particularly prone to negative coping strategies, such as binge drinking and smoking [11]. Being out of work at a young age also increases the risk of decreased income later in life [14], which influences mortality negatively [16]. The decreased relative risk for older adults without full-time employment may also be related to the fact that older people are more susceptible to disease. In addition, changes in LMS category towards weaker labour attachment later in life are relatively common. However, we have no information on changes in LMS category status after baseline, which means that the decreased relative risk during follow-up may partly be due to misclassification.

While other studies have indicated an association between unemployment and sickness absence and mortality in both young men and women [7, 14], our analyses did not show a statistically significant association between sick leave/rehabilitation allowance or unemployment benefits and increased mortality risk in women. One possible explanation for this may be that a relatively large proportion of the sickness absence among young women in Norway is caused by musculoskeletal, pregnancy, and birth-related conditions, which are usually not lethal [26]. In addition, fewer women than men belonged to the reference category full-time work, which led to low statistical power and wide CIs. Since the health survey was carried out in 1987–1988, a larger proportion of Norwegian women have entered the labour force [27]. Thus, the group of women who are not employed now may be somewhat different and may have different mortality risks.

The present study showed an increased mortality risk for women in the oldest age groups with the LMS category no paid work/homemaker. Relatively few studies have explored the health and mortality risks of homemakers, but the ones that do exist have showed contrary findings, and none examined age-varying associations between being a homemaker and mortality [28–31]. A possible reason for the increased mortality risk among our oldest homemakers may be their reduced retirement pension, which affects health and mortality negatively [32].

An association between low education level and non-employment was found in this study. Low education was particularly evident for persons on disability pension and is in line with previous observations [4–6, 20]. Social inequality in work exclusions may be related to several factors, including poorer health and/or working conditions [33]. Furthermore, work exclusions are not only unevenly distributed socioeconomically, but also geographically [34], and in the years before and after the baseline survey in the Finnmark cohort study, a resource crisis in commercial fisheries contributed to considerable workforce reductions in this rural county.

### Strengths and limitations

All residents in Finnmark County aged 40–62 years as well as a 20% representative sample of residents aged 30–39 years were invited to participate in the baseline study, and strengths of the study are the high attendance [13], the fact that all participants completed questionnaire 1, and the 30 years of follow-up mortality data. Only 73% and 79% of participants completed questionnaires 2 and 3, respectively, which can be seen as a weakness of the study. However, our analyses that compared associations among participants with complete data in the FPS model and those with data on sex, age, marital status, LMS category, and smoking showed small differences. This is in line with former studies that examined the possibility of distortions in associations and concluded that these distortions were not of sufficient magnitude substantially to bias estimates [13, 19].

As far as we know, no other studies have examined the age-varying associations between LMS categories and mortality. The examination of age-varying associations can be seen as a strength of the study, because an analysis with standard time-constant estimates for men who were not full-time employees and women with disability pension could have led to an underestimation of mortality risk in younger participants and an overestimation of risk in older participants [24].

One possible weakness of the study may be related to the inclusion of covariates. According to VanderWeele [35], variables which cause the exposure and/or outcome should be included as covariates, while variables which are mediators on the pathway between the exposure and outcome should not be included. However, for some of the considered covariates in the present study, it is difficult to ascertain whether they are causes or mediators. One example is smoking, which may both be a cause of increased mortality risk and a result of coping after experiencing non-employment.

The variable LMS category has strengths and weaknesses. Few persons aged 30–62 years with a LMS category of disability pension change their category, unlike those with other LMS categories, who change relatively commonly, and more than one measurement of LMS category could have given more robust estimates. The category ‘sick leave/rehabilitation allowance’ consists of different groups, such as persons on short-term sick leave, long-term sick leave, and rehabilitation allowance, while the category ‘unemployment’ consists of both people with short-term and long-term unemployment, which are groups which may have a different mortality risk. On the other hand, relatively few participants belonged to these categories, and splitting it would have led to loss of statistical power.

## Conclusions

The present study showed an association between low education level and non-employment.

Men with part-time work, sick leave/rehabilitation allowance, unemployment benefits, or disability pension had increased mortality risk compared with full-time employees, but these findings were restricted to ages below 60–70 years, varying with LMS category. For women, excess mortality was linked to disability pension in the younger age groups, and to the LMS category no paid work/homemaker for older age groups. Our findings indicate that the increased mortality risk for certain LMS categories is partly explained by health, pre-existing illnesses, and health-related behaviour and partly by other factors, such as social network and economic factors.

## References

[1] de la Fuente A. New measures of labour market attachment. Report no. ISSN 1977-0316. Luxembourg: Eurostat, 2011.

[2] Gustafsson K Aronsson G Marklund S , et al. Peripheral labour market position and risk of disability pension: a prospective population-based study. BMJ Open 2014;4:e005230. DOI: 10.1136/bmjopen-2014-005230 PMC4139627 25142263

[3] Eurostat. Glossary: People outside the labour force. See https://ec.europa.eu/eurostat/statistics-explained/index.php?title=Glossary:People_outside_the_labour_force (2020, accessed 5 April 2022).

[4] Gjesdal S Maeland JG Svedberg P , et al. Role of diagnoses and socioeconomic status in mortality among disability pensioners in Norway – a population-based cohort study. Scand J Work Environ Health 2008;34:479–482. 2009/01/13. DOI: 10.5271/sjweh.1286 19137210

[5] Polvinen A Laaksonen M Gould R , et al. Socioeconomic inequalities in cause-specific mortality after disability retirement due to different diseases. Scand J Public Health 2015;43:159–168. 2014/12/17. DOI: 10.1177/1403494814562597 25504585

[6] Van der Wel KA Dahl E Birkelund GE. Employment inequalities through busts and booms:the changing roles of health and education in Norway 1980–2005. Acta Sociologica 2010;53:355–370. DOI: 10.1177/0001699310380063

[7] Roelfs DJ Shor E Davidson KW , et al. Losing life and livelihood: a systematic review and meta-analysis of unemployment and all-cause mortality. Soc Sci Med 2011;72:840–854. DOI: 10.1016/j.socscimed.2011.01.005 21330027 PMC3070776

[8] Dunlavy AC Juárez S Rostila M. Employment status and risk of all-cause mortality among native- and foreign-origin persons in Sweden. Eur J Public Health 2018;28:891–897. 2018/06/04. DOI: 10.1093/eurpub/cky090 29860314

[9] Gjesdal S Svedberg P Hagberg J , et al. Mortality among disability pensioners in Norway and Sweden 1990–96: comparative prospective cohort study. Scand J Public Health 2009;37:168–175. DOI: 10.1177/1403494808100937 19179451

[10] Karlsson NE Carstensen JM Gjesdal S , et al. Mortality in relation to disability pension: findings from a 12-year prospective population-based cohort study in Sweden. Scand J Public Health 2007;35:341–347. DOI: 10.1080/14034940601159229 17786796

[11] Roelfs DJ Shor E Blank A , et al. Misery loves company? A meta-regression examining aggregate unemployment rates and the unemployment-mortality association. Ann Epidemiol 2015;25:312–322. 2015/03/22. DOI: 10.1016/j.annepidem.2015.02.005 25795225 PMC4397178

[12] Gjesdal S Ringdal PR Haug K , et al. Mortality after long-term sickness absence: prospective cohort study. Eur J Public Health 2008;18:517–521. 2008/03/12. DOI: 10.1093/eurpub/ckn010 18332039

[13] Fylkesnes K Jakobsen MD Henriksen NO. The value of general health perception in health equity research: a community-based cohort study of long-term mortality risk (Finnmark cohort study 1987–2017). SSM – Population Health 2021;15: 100848. DOI: 10.1016/j.ssmph.2021.100848 PMC8237603 34195347

[14] Helgesson M Johansson B Nordqvist T , et al. Sickness absence at a young age and later sickness absence, disability pension, death, unemployment and income in native Swedes and immigrants. Eur J Public Health 2015;25:688–692. 2015/01/31. DOI: 10.1093/eurpub/cku250 25634955 PMC4512957

[15] Gjesdal S Mæland JG Hagberg J , et al. Socioeconomic inequalities and mortality among disability pensioners in Norway – a population-based cohort study. Norsk Epidemiologi 2007; 17:29–35.

[16] Wallman T Wedel H Johansson S , et al. The prognosis for individuals on disability retirement. An 18-year mortality follow-up study of 6887 men and women sampled from the general population. BMC Public Health 2006;6:103. 2006/04/25. DOI: 10.1186/1471-2458-6-103 16630360 PMC1459134

[17] Westlund K Søgaard AJ. Helse, livstil og levekår i Finnmark. Resultater fra Hjerte-karundersøkelsen i 1987–88. Finnmark III. 1993. Tromsø, Norway: Universitetet i Tromsø, 1993.

[18] Bjartveit K Foss PO Gjervig T , et al. The Cardiovascular Disease Study in Norwegian Counties. Acta Medica Scand 1979; 634:1–70. 293122

[19] Fylkesnes K Førde OH. Determinants and dimensions involved in self-evaluation of health. Soc Sci Med 1992;35:271–279. 1519079 10.1016/0277-9536(92)90023-j

[20] Fevang E Røed K. Veien til uføretrygd i Norge. 2006. Oslo: Ragnar Frisch Centre for Economic Research, 2006.

[21] Royston P Lambert P. Flexible Parametric Survival Analysis Using Stata: Beyond the Cox Model. College Station, Texas: Stata Press, 2011.

[22] Kleinbaum DG Klein M. Survival Analysis: A Self-Learning Text, 2nd ed. New York: Springer, 2005.

[23] Thiébaut AC Bénichou J. Choice of time-scale in Cox’s model analysis of epidemiologic cohort data: a simulation study. Stat Med 2004;23:3803–3820. 2004/12/08. DOI: 10.1002/sim.2098 15580597

[24] Dickman P. Graphing the HR as a function of time. See http://pauldickman.com/software/stata/sex-differences-cox/#graphing-the-hr-as-a-function-of-time (accessed 6 October 2021).

[25] Modini M Joyce S Mykletun A , et al. The mental health benefits of employment: results of a systematic meta-review. Australas Psychiatry 2016;24:331–336. DOI: 10.1177/1039856215618523 26773063

[26] Berge C. Uendret sykefravær siden 2001. Samfunnsspeilet 2010;24:16–23.

[27] Statistics Norway. Fakta om likestilling. See https://www.ssb.no/befolkning/faktaside/likestilling (accessed 28 April 2022).

[28] Passannante MR Nathanson CA. Female labor force participation and female mortality in Wisconsin 1974–1978. Soc Sci Med 1985;21:655–665. DOI: 10.1016/0277-9536(85)90205-9 3877345

[29] Ahs AM Westerling R. Mortality in relation to employment status during different levels of unemployment. Scand J Public Health 2006;34:159–167. 2006/04/04. DOI: 10.1080/14034940510032374 16581708

[30] Roos E Lahelma E Saastamoinen P , et al. The association of employment status and family status with health among women and men in four Nordic countries. Scand J Public Health 2005;33:250–260. DOI: 10.1080/14034940510005680 16087487

[31] Arber S Lahelma E. Inequalities in women’s and men’s ill-health: Britain and Finland compared. Soc Sci Med 1993;37:1055–1068. DOI: 10.1016/0277-9536(93)90440-F 8235738

[32] Shahidi FV Parnia A. Unemployment insurance and mortality among the long-term unemployed: a population-based matched-cohort study. Am J Epidemiol 2021;190:2124–2137. DOI: 10.1093/aje/kwab144 33997895

[33] Marmot M Friel S Bell R , et al. Closing the gap in a generation: health equity through action on the social determinants of health. Lancet 2008;372:1661–1669. DOI: 10.1016/S0140-6736(08)61690-6 18994664

[34] Krokstad S Westin S. Disability in society-medical and non-medical determinants for disability pension in a Norwegian total county population study. Soc Sci Med 2004;58:1837–1848. 2004/03/17. DOI: 10.1016/s0277-9536(03)00409-x 15020001

[35] VanderWeele TJ. Principles of confounder selection. Eur J Epidemiol 2019;34:211–219. 20190306. DOI: 10.1007/s10654-019-00494-6 PMC6447501 30840181

